# Patients’ Experiences of Web- and Mobile-Assisted Group Therapy for Depression and Implications of the Group Setting: Qualitative Follow-Up Study

**DOI:** 10.2196/mental.9613

**Published:** 2018-07-11

**Authors:** Raphael Schuster, Sophia Sigl, Thomas Berger, Anton-Rupert Laireiter

**Affiliations:** ^1^ Center for Clinical Psychology, Psychotherapy and Health Psychology Department of Psychology University of Salzburg Salzburg Austria; ^2^ Faculty of Psychology University of Vienna Vienna Austria; ^3^ Department of Clinical Psychology and Psychotherapy University of Berne Berne Switzerland

**Keywords:** internet, computer-assisted therapy, smartphone-assisted therapy, blended therapy, cognitive behavioral therapy, depression, therapeutic process, working alliance

## Abstract

**Background:**

Blended group therapy combines group sessions with Web- and mobile-based treatment modules. Consequently, blended group therapy widens the choice within blended interventions at reasonable costs. This is the first qualitative study on blended group therapy.

**Objective:**

The objective of this study was to investigate the patient-centered feasibility of blended group therapy for major depression, with special emphasis on the fit and dynamic interplay between face-to-face and internet-based elements.

**Methods:**

A total of 22 patients who had a variety of experiences through participating in one of the two blended group therapy interventions were interviewed following a semistructured interview guide. In-depth interviews were analyzed by three trained psychologists, using thematic analysis and a rule-guided internet-based program (QCAmap). The transcript of the interviews (113,555 words) was reduced to 1081 coded units, with subsequent extraction of 16 themes.

**Results:**

Web- and mobile-based elements were described as a treatment facilitator and motivator, increasing the salience and consolidation of cognitive behavioral therapy materials, resulting in in- and inter-session alignment to the treatment. Additionally, patients valued the option of intimate Web-based self-disclosure (by lateral patient-therapist communication), and therapists were provided with tools for between-session monitoring and reinforcement of exercising. In this context, group phenomena seemed to back up therapists’ efforts to increase treatment engagement. The dissonance because of noncompliance with Web-based tasks and the constriction of in-session group interaction were considered as possible negative effects. Finally, issues of tailoring and structure seemed to fulfill different preconditions compared with individual therapy.

**Conclusions:**

Blended group therapy constitutes a structured and proactive approach to work with depression, and the integration of both modalities initiates a beneficial interplay. Results support the patient-centered value of blended group therapy and provide the first insight into blended group therapy’s role in fostering therapeutic treatment factors. However, potential negative effects should be considered carefully.

## Introduction

Depression, as a prevalent mental disorder, presents a relevant public health concern and incurs substantial economic costs on public health care [1]. Among others, research priorities in mental health care include the development and evaluation of internet-based and eHealth treatments (eg, the development of new treatments and adherence to these treatments) [2].

Internet-based interventions (syn. Web-based interventions and computer-supported interventions) feature different degrees of therapist support. On a basic level, unguided stand-alone treatments display wide reach at most reasonable costs. At the same time, therapist guidance can help to improve treatment effects and reduce dropout rates significantly [3,4]. However, certain characteristics of remotely operating guided and unguided interventions seem to decelerate and limit dissemination. Here, the lack of personal contact, restricted management of comorbidity or crisis, as well as the stakeholders’ attitude and legal restrictions act as prominent barriers [5]. To address these factors, internet-based interventions can be merged with classic face-to-face therapy, resulting in the treatment format of a blended therapy (syn. Web-, computer-, and mobile-assisted therapy).

Blended therapy may be considered as any combination of Web-, mobile-, or technology-based application with classic face-to-face therapy, resulting in possible savings of therapist time (efficiency) or treatment intensification (efficacy) [6]. In this context, feasibility studies investigate the utility of blended interventions for the in- or between-session treatment of common mental disorders such as anxiety or depression [7,8]. Other studies aimed at reducing the number of personal sessions needed for treatment [9-11]. These reductions should result in postulated increases of cost-efficiency. Even though some blended interventions are designed to intensify treatment [12], corresponding literature remains scarce [13-16].

Besides individual therapy, the blended format has been applied in psychological group treatments (blended group therapy, bGT). Group therapy has many possible applications, and various guidelines recommend this treatment. For example, the National Institute for Health and Care Excellence [17] recommends group cognitive behavioral therapy (CBT) for people who decline other low-intensity psychosocial interventions such as internet-based interventions. Previously, the use of bGT for the treatment of frequent anxiety disorders, such as social phobia [18] or generalized anxiety disorder [19,20], has been tested. Regarding depression, our research group developed and tested a series of low-threshold and stigma-free interventions for subclinical, as well as clinical, depression [6,15]. Aside from high to very high treatment effects on depressiveness, in- and inter-session computer support was consensually described as a therapeutic factor, contributing to the treatment intensification [6,15].

Regarding patients’ experiences with blended therapy, studies exist in the form of posttreatment surveys and semistructured interviews. Based on a Delphi study, van der Vaart et al [21] assessed the possible benefits and drawbacks of blended individual therapy. The perceived benefits of applied internet-based components concerned the improvement of therapy-related self-management, flexibility of treatment complementation, and reduced traveling time. On the other hand, half of the involved patients feared that the patient-therapist bonding could be weakened, and difficulties or indistinct matters could be harder to communicate. In addition, the majority agreed that the blended format is not suitable for all patients and that treatment should be tailored to individual needs. In a qualitative study, Ly et al [22] examined patients’ experiences with a mobile phone behavioral activation app, combined with short therapist contacts (maximum 20 min/week). Owing to its permanent availability, a subgroup of patients described the app as a treatment facilitator and motivator. At the same time, most interviewees agreed that the app was supportive but not sufficient for the treatment of depression, and many participants of the minimal contact intervention would have liked more personal contact. These findings are in line with a qualitative study on patients’ motivation to persist with a blended intervention (internet-based intervention + short face-to-face consultations) [23]. According to the authors, patients were motivated to persist with the intervention when their need for relatedness was satisfied. In this context, Self-Determination Theory (SDT) [24], which postulates relatedness, competence, and autonomy being the three relevant agents for intrinsic motivation, served as the underlying theoretical framework. In a second evaluation, internet-based components were perceived as a knowledge source, providing a structured approach to work with depression [25]. Moreover, enhanced patient skills and beneficial effects on the therapy process were reported by Mansson et al [7], who described internet-based content as a source of information and a memory aid. Some patients reported fostered treatment engagement and reduced avoidance of feared situations. Furthermore, the therapists involved suggested that the structure provided by internet-based components might counteract the tendency to drift away from evidence-based treatment rationales (therapist drift) [26].

To sum up, patients’ experiences contribute to the understanding of new treatments and are crucial in the development of blended interventions. While blended individual therapy for major depression has been evaluated several times, bGT has not been the objective of intensive research yet. Thus, one might wonder if the preconditions of bGT are identical to blended individual therapy and classic group therapy, or if the dynamic interplay between therapeutic groups and technology results in new phenomena or in setting-specific benefits and drawbacks. Based on the follow-up interviews of two recently conducted clinical studies for major depression, this study aims to shed light on the patient-centered evaluation of bGT.

## Methods

### Study Approach

This study was conducted as a follow-up to two recently conducted clinical trials in Salzburg and Vienna, Austria (German Clinical Trial Registration, DRKS: DRKS00010894; DRKS00010888). Both trials were approved by the Local Ethics Committees (Ethical Review Board University of Vienna, Ref-Nr: 00194; University of Salzburg, Ref-Nr: EK-GZ:18/2016). The studies aimed to test the feasibility, usability, and effectiveness of two newly developed bGT interventions for the outpatient treatment of mild to moderate depression and comorbid anxiety.

### Interventions

Both treatments entailed psychoeducative techniques, combined with either self-management or behavioral activation and in- and between-session media, Web and mobile support. Sessions were held using a double trainer format by 7 trained and supervised psychologists. Group sessions (90 minutes), Web- and app-based homework tasks, and remote therapist feedback alternated within each week. Patients received feedback on preparatory Web-sessions. Each Web-session was followed by a personal group session, and the mobile application enhanced the transfer of each week’s topic (eg, acceptance). The first intervention (A) was based on resource-oriented techniques, positive psychology, and self-management and entailed 7 face-to-face group therapy sessions. Multimedia presentations, e-learning (videos and worksheets), an unguided group-chat, as well as a remote patient-therapist communication complemented the group intervention [15]. The second intervention (B) was based on the principles of the Acceptance and Commitment Therapy (ACT) [27] and behavioral activation [28]. Except for the unguided group chat and an additional mobile phone-based diary app, both interventions featured identical treatment elements. The second intervention was operated by the Minddistrict platform [29]. The first and last authors of this study participated in planning and evaluating both trials.

### Trial Context and Involved Patients

For trial recruitments, we selected multiple strategies, comprising a newspaper article, Web-based advertisement, as well as handing out flyers in public health centers, general practitioner practices, and frequently visited public areas. All those interested were invited to visit the study Web page and fill out an internet-based participation form.

Based on the independent clinical interviews [30], patients were eligible to participate in one of the clinical trials if they suffered from major depressive disorder (MDD), dysthymia, or comorbid anxiety (or a combinations of these two or more conditions simultaneously), had not undergone psychotherapy, took medication constantly for at least3 months, were aged 18-65 years, possessed a personal computer and a mobile phone, had access to the internet, and were fluent in German. According to the clinical judgment, participants were excluded if they suffered from severe depression, severe anxiety disorder, bipolar disorder, substance abuse, severe psychiatric and psychotic conditions, schizoaffective disorder, or suicidal ideation.

### Interview Recruitment and Data Collection

In this study, all patients of both trials received a written invitation to participate starting within 1-8 months after treatment had ended. The recruitment was continuous, and we strived to include the highest possible number of participants with the widest range of personal experience. Therefore, an incentive of 20 Euro was offered for participation, and in the invitation, a special emphasis was placed on including less positive experiences as well. Nonresponders were repeatedly contacted by a second follow-up invitation. Among 53 eligible patients, 22 agreed to participate in the qualitative evaluation (response rate= 41.5%). We considered this response rate and the gained sample composition sufficient for depicting a variety of different experiences. Participants’ age ranged from 21 to 64 years (mean 36.2, SD 11.6 years). Owing to strategical invitations and prompts, we were able to include one intervention withdrawal (33% of all withdrawals), as well as a good proportion of relatively unsatisfied patients (3 patients, 50% of all unsatisfied patients; according to the Client Satisfaction Questionnaire, CSQ [31]) and 7 patients with comparably low system usability ratings (70% of all low system usability ratings; according to the System Usability Scale, SUS [32]). Thus, the SUS ratings represented a more critical sample than the original population. The reduction of depressiveness also varied in the sample, including one deterioration and 6 nonresponders. Table 1 presents a detailed sample description.

Retrospective semistructured in-depth interviews were conducted in equal parts by the first (RS) and second (SS) authors between 3 and 9 months after participation (M=4.1 months). Both psychologists were blinded to the outcome of patients at the time of interviews. Audiorecorded interviews were based on a 16-question interviewing guide (Textbox 1 and Multimedia Appendix 1) and lasted between 25 and 70 minutes (M=46 minutes). Verbal informed consent was obtained at the beginning of each interview. The content of the interview guide was based on prior blended therapy research [21-23,25]. Basic questions concerned the expectations and motivation to participate in the clinical trial, as well as perceived advantages and disadvantages of bGT. Further questions regarded experience-related process aspects of bGT such as perceived structure, intensity, and effectiveness. Finally, patients were invited to share memories of the treatment and provide feedback on potential improvements.

### Data Analysis

Recorded interviews were transcribed by the second author, together with an independent, additional trained psychological assistant. In this study, we selected the thematic analysis as an extraction method [34]. In this approach, meaningful patterns are pinpointed by a variety of initial codes, which in turn, get bundled into subcategories and subsequently result in designated main themes. By synthesizing a deductive and theory-driven frame (cf. interview guide) with an inductive and data-driven extraction process, this process of content extraction can be classified as hybrid [35]. This strategy allows a systematic allocation of observed phenomena within existing concepts, while it preserves the transmissibility for new phenomena.During sequential data analysis, the written transcripts (113,555 words) were repeatedly read by the second author and the independent psychological assistant. For data extraction, the transcripts were uploaded to the QCAmap platform (QCAmap [36]), which provides standardized rule-guided qualitative categorization and allows internet-based code extraction. The principal code system was created by the second author (SS) and revised by the independent psychological assistant and the first author (RS), with the possibility to adapt or rename a given unit. When applicable, a valence was assigned to the codes (positive vs negative or advantage vs disadvantage). As not all codes fitted into this scheme, a good proportion of codes remained neutral. After 25% of the text had been coded, the principal extraction and independent revision of meaning units were stopped, and all three analysts determined preliminary coding units to work with. Subsequently, the entire text was analyzed, resulting in 1081 coded passages. In the next step, meaning units were extracted and grouped into subthemes, which in turn were validated by revisiting corresponding text passages. After this, subthemes were refined by the first and second authors, together with the independent psychologist assistant, to create a coherent wording and structure. Finally, refined subthemes were related to main themes, which often corresponded to the predefined topics of the applied interview guide.

**Table 1:** Characteristics of interviewed patients (N=22).

Number	Age (years)	Gender	Employment	Diagnosis	Comorbidity	Diff CES-D^a^ posttreat^b^	CSQ^c^	SUS^d^	Intervention
1	21	Female	Student	F32.0 MDD^e^, mild	—	−9	✓	✓	B^f^
2	37	Female	Part-time	F32.0 MDD, mild	—	−9	✓	✓✓	B
3	35	Male	Full-time	F32.1 MDD, moderate	F41.1	−3	✓✓	✓	A^g^
4	45	Female	Unemployed	F32.1 MDD, moderate	F34.1+F40.2	−15	✓✓	✓	A
5	33	Female	Full-time	F41.1 GAD^h^	F32.0	−7	✓	✓✓	B
6	31	Female	Full-time	F32.0 MDD, mild	—	−28	✓✓	✓	A
7	56	Female	Full-time	F41.0 Panic disorder	—	+9	✓✓	✗	A
8	24	Female	Part-time	F32.1 MDD, moderate	F34.1+F41.0	+/−0	✓	✓	A
9	27	Male	Student	F32.1 MDD, moderate	—	−17	✓✓	✓	B
10^i^	28	Female	Marginally	F32.1 MDD, moderate	—	−13	✓✓	✓	B
11	28	Male	Full-time	F32.0 MDD, mild	—	−5	✓✓	✓	B
12	47	Female	Unemployed	F32.1 MDD, moderate	—	−11	✓	✓	B
13	45	Male	Full-time	F33.0 MDD, recurrent	—	−14	✓✓	✓	A
14	24	Male	Student	F40.1 Social phobia	F33.4	−5	✗	✓	B
15	40	Female	Full-time	F32.0 MDD, mild	—	−3	✗	✗✗	B
16	34	Female	Full-time	F32.0 MDD, mild	—	−11	✓	✗	B
17	26	Female	Part-time	F32.1 MDD, moderate	F41.1	−22	✓✓	✓	B
18	46	Male	Full-time	F32.0 MDD, mild	—	−13	✓✓	✗	B
19	24	Female	Part-time	F32.0 MDD, mild	F40.1	−2	✓✓	✗	B
20	64	Female	Full-time	F33.0 MDD, recurrent	—	−10	✗	✓	A
21	50	Female	Full-time	F33.1 MDD, recurrent	—	−11	✓✓	✗	A
22	32	Male	Part-time	F33.0 MDD, recurrent	—	−9	✓	✗	A

^a^CES-D: Center for Epidemiological Studies Depression Scale [33].

^b^Diff CES-D posttreat: difference in depressiveness from pretreatment to posttreatment (✓✓, “good”; ✓, “okay”; ✗, “poor”; ✗✗, “awful”).

^c^CSQ: Client Satisfaction Questionnaire [31] (✓✓ >27; ✗ <24).

^d^SUS: System Usability Scale [32]

^e^MDD: major depressive disorder.

^f^B: Acceptance and Commitment Therapy-based intervention.

^g^A: resource-oriented intervention.

^h^GAD: generalized anxiety disorder.

^i^Withdrawal, diagnoses according to the International Statistical Classification of Diseases and Related Health Problems 10^th^ Revision.

**The short version of the applied interview guide. The full version of the interview guide can be found in Multimedia Appendix 1.**

1. What were your expectations regarding the treatment?
2. How would you describe the treatment?
3. How would you describe the treatment’s format (comprising group and media components)?
4. How did you use the online platform?...the videos?...the app?
5. How did you experience the group? What did you experience positively/negatively?
6. What are particular (dis-) advantages of the computer-supported / mobile-supported format?
7. Regarding modern media (app + platform + in-session), what would you change if you could?
8. How would you evaluate the blended format?
9. How did technology influence the group / group interaction?
10. How would you describe the treatment in terms of structure and flexibility?
11. How did the therapists handle technology?
12. How useful is the application of modern media in psychological groups?
13. How would you describe the treatment’s intensity?
14. Was there anything particularly difficult to motivate yourself for?
15. What impact did the intervention have on your thoughts, feelings, plans, and behaviour?
16. Is there anything (important) you would like to add to this conversation?

## Results

### Analysis Results

Based on the 1081 coded passages, the analysis of 22 interview transcripts identified 3 generic and 13 setting-specific themes as follows: (1) patients’ expectations; (2) general appraisal of the treatment; (3) suitability for use, as well as appraisal of the blended format, disadvantages of the blended format, and structure and tailoring; (4) self-disclosure and internet-based reinforcement; (5) dynamic interplay of group and technology; (6) negative effects; (7) design, treatment discontinuity, and motivation; (8) workload; (9) enhanced psychoeducation and monitoring; (10) quality of treatment; (11) appraisal of single tasks; (12) data safety and technical issues; and (13) principles of action or therapeutic factors. Owing to the quantity and breadth of results, we will only address the first 2 generic and 6 treatment-specific themes (Table 2), while the remaining results will be published in the near future. In order to establish a dramaturgical thread, this study focuses primarily on generic themes (2 of 3) and aspects that contribute to the basic understanding of bGT; however, the forthcoming study will be dedicated to differential treatment aspects and the optimal design of bGT. Even though not entirely consistent, the division between generic and specific themes (Table 2) refers to topics that specifically relate to blended therapy and the topics of a more general experience with the undergone treatment.

### Patients’ Expectations

Patients’ treatment expectations and motivations entailed several aspects in equal parts. Most patients either mentioned the need for psychological depression treatment, or they were searching for help to deal with critical life events, including incipient signs of burnout, panic, or other crises. Three patients mentioned prior stays at psychiatric clinics and the urge to counteract current relapse tendencies. Other 3 patients were looking for alternatives to conventional psychotherapy or medication. In addition, 5 of the patients considered the group as a place for exchange and mutual support. Contrarily, 2 patients reported initial concerns about the group setting. Furthermore, a minor number of comments pertained to the novelty and curiosity of the treatment or the desire to work with a structured approach and receive tools against depression.

Not too many [expectations]. I have suffered from depression for several years. I wanted to find better ways of dealing with it and also to actively do something against it without the need for medication.

B5

To somehow untangle a knot. My children consume a lot of my attention and I also think a lot about my own childhood. Sometimes I simply don’t think I can manage it. So I was looking for a solution.

B4

I was diagnosed with depression and I thought, [...] okay that’s an easy way to seek psychological treatment. I was also curious and interested in learning ways to cope with it.

B11

The first idea was to get in contact with people. Since moving here I have felt quite well. But, after a while, I realized that I was relapsing into old habits that were not any good. So, I hoped for some input. [...] A few years ago, I stayed in a psychiatric clinic for some weeks.

B14

**Table 2:** Patients’ main themes, subthemes, and frequent codes.

Main themes and subthemes			Frequent codes
**Generic theme**			
	**Expectations**		
		Help	Treatment; alternative treatment; support with life events
		Group	Exchange; self-disclosure; mutual support; being connected to others; concerns about the group
		Intervention	Novelty and curiosity; tools against depression; a structured approach to addressing problems
	**Appraisal of treatment**		
		Group	Cohesive entity; atmosphere; self-disclosure and discussion; preference over the platform
		Intervention	The shift of modalities; structure and composition; practical use
		Experience	Useful insights; positive over all experience
**Theme specifically related to the investigated blended format**			
	**Evaluation of blended format**		
		Advantages	Contemporary or innovative or engaging approach; transparency or flexibility of approach; blending supports treatment; reminder; enhances consolidation; helps group interaction
		Disadvantages	Diverging preferences; the importance of group; low usability; concerns regarding format; workshop-like
	**Evaluation of blended components**		
		Advantages platform	Provides information; increases information processing; interactive; practical; clear structure; saves time for the group
		Advantages app	Reminder; transfer; documentation of mood or activities; awareness; insight or understanding
		Advantages in-session media	Guides therapists; provides information; supports therapists
		Disadvantages of all	Low usability of app or platform; does not suit everyone; too little tailoring of reminders; a sense of obligation or annoyance; discouragement due to noncompliance; suboptimal trainer behavior
	**Structure and tailoring**		
		Structure	Positive appraisal of the structure; guiding thread; a systematic approach to work with; unmet needs
		Blending	Increased individuality; blending leads to the structure; risk of overload
	**Self-disclosure and internet-based reinforcement**		
		Reinforcement	The increased presence of therapist; feedback motivates; personal feedback; support with exercises
		Self-disclosure	The extra path for self-disclosure; extra information for the therapist; group hinders self-disclosure
	**Dynamic interplay of group and technology**		
		Preview or rework	Preview; rework; alignment; group increases compliance with Web-based elements
		Smoothing effect	Reduces undesired effects; group drift; domination
	**Negative effects**		
		Therapist level	Therapist’s time management; therapist’s patient management
		Patient level	Restricted group interaction; noncompliance results in dissonance; low added value
		Technology	Design or technical or data safety issues

### Overall Experiences With the Treatment

Here, we describe experiences with most salient treatment aspects, as well as appraisals of its most valued elements. In total, we received 52 comments and a 5:1 ratio describing positive to negative treatment features. Frequently, the group was described as a cohesive entity, providing an amicable atmosphere for exchange, self-disclosure, and discussion. In this context, 2 patients also expressed their preference for the group over the Web-based platform. The second popular category consisted of positive comments on the interplay of modalities (eg, psychoeducation or discussion and exercising), structure and composition of the elements, as well as their practical use. The third category contained comments on interesting or useful insights and positive overall experience. These categories were followed by 4 other medium-frequency meaning units. While the role of the therapists was acknowledged in one unit, another unit was dedicated to the compactness and transparency of the treatment. In addition, 4 patients pointed out that everyone was able to put special emphasis on personally relevant treatment aspects, and 3 patients described the treatment as a low-threshold intervention, which does not fit the concept of conventional psychotherapy. Furthermore, negative experiences regarded ACT as a demanding concept (2) and the group as a demanding setting (1). In fact, 4 patients described the treatment as too short, too fast, or too structured.

It was thrilling and interesting for me. I don’t particularly like these group discussion situations. [...] But at same, you learn that you aren’t the only one. [...] For me it was more of a workshop rather than a therapy.

B6

It was a nice mix of talking and practical exercises. [...] And it was in a group [...]. This was quite nice, because I frequently feel as if no one understands me. [...] I found a friend there and we are still in contact.

B12

I think it [the therapy] was arranged well. I can’t say that everything suits everyone, but you can pick out things that suit.

B18

Sometimes it [the in-session presentation] whizzed by so fast and I did not manage to keep up. But still, it was interesting and the two [therapists] did a great job. [...] The online part also worked well, with interesting videos. It was quite interactive.

B15

### General Evaluation of the Blended Format

As central success criteria for the new treatment, users’ general evaluation of the blended format can be regarded as the overall experience with the Web- and mobile-supported interventions, as well as the conceptual validation of their most important features. In this context, we identified 71 (82%) positive and 16 (18%) critical sections. Participants frequently described the treatment as a contemporary and innovative approach, entailing an engaging and transparent treatment course. The majority of patients appreciated the supportive role of technology, and the blended format was reported to act as a reminder and to increase the consolidation of treatment materials. In addition, some patients described it as a treatment intensifier. The Web-based platform was frequently associated with treatment flexibility and increased self-management. Many patients commented on the beneficial phenomena regarding the group’s dynamic interplay with technology. In this regard, a corresponding in-depth analysis is being provided in the next section. Last but not least, many comments constituted generic appraisals of the blended format.

I like the idea of merging those methods.

B16

I think media can improve the treatment.

B22

I only find advantages in all three of them [group media, platform, and app]. I think it’s good, because it’s the way of the future.

B17

I have psychotherapy experience, but I definitely prefer this format.

B21

It makes sense. People engage freely and relate something positive and relaxing to it. If you use it for psychological work, then this “couch-interview-dependency-factor” disappears.

B4

I think it’s a good idea, because the platform was supportive. In the sessions we talked about its content—that’s a good combination.

B7

The combination was nice. I understand things better when I see them. For example, I still remember this short clip with the dog. Looking at it from this point of view was nice as I could recognize myself in it.

B8

I like this approach a lot. It means autonomy. You do not only participate in the group sessions. If you are interested, you can prepare yourself or rework something. So, you stay in the flow.

B13

Critical statements of the overall experience ranged from personal preferences, the importance of group experience and usability aspects, to serious concerns about the application of technology in psychotherapy. Three patients described the treatment’s workshop-like character as unusual or unfitting.

It is okay for me. It is modern. I’m just not really the “online tool study” type.

B18

To me, personal contact is more important, than watching videos or letting something else wash over me.

B2

I found it way too complex. With these videos and then the app—so many platforms and I was constantly searching for my password. For me it was simply too complicated.

B15

I wonder why psychology deals that much with technology. Due to my prior therapy experience, I’m sceptical when it comes to standardisation and thinking inside boxes. It was somehow disconcerting for me.

B20

### Advantages and Disadvantages of Blended Components

In this section, patients shared their thoughts and experiences with the blended components—the Web-based platform, in-session media use, and mobile apps. Many comments reflected on the aspects of usability, facilitation, and habituation. As the first component, the Web-based platform was described as a useful and economic source of information. In this context, weekly presented videos were described as having an interactive, illustrating, entertaining, and explanatory quality. In addition, the platform’s practical handling was highlighted in terms of the information content and clarity, individual pacing, fostered information processing, and time savings for group sessions.

In the first place it is guiding and deepening and particularly timesaving. [...] Because if you’d work on it during the group meetings a lot of group time would be lost. In that sense, it’s good to receive it in advance and then discuss it with the group.

B20

I liked the online platform most, perhaps because everything is arranged clearly on a big screen.

B15

For me it [the platform] was a ritual: I entered the password to logon, and I was in. And that’s the time I took for myself. And then I simply work on it.

B6

You can assess the content whenever you need to. [...] So, one is able to work at his/her own pace—that’s the great thing.

B4

This is mere speculation, but maybe computer support increases the effectiveness of the treatment.

B16

The mobile app was primarily described as a reminder and transfer facilitator. In addition, it supported the close documentation of mood or activities and helped increase awareness. Furthermore, some participants reported better understanding of the problematic behavior or (emotional) triggers.

To assess one’s mood—as in a diary—that’s a fantastic idea, because you can track it over a long period of time. And I think that helps find out, if certain things help, or if they don’t. You could also use a sheet of paper, but the app provides more features.

B9

You have to document your mood [...] and in the end it sums it up as a time line. That’s pretty nice. This might help in identifying triggers and things that repeatedly put you down.

B20

You get reminded of the awareness. It’s not like writing a diary: what happened today in your life? Instead it’s really: what do you feel right now. That’s a nice input, because I would not have come to ask myself that profoundly.

B10

The app was great to receive reminders and everything. It was interesting, quite rapidly you don’t need the reminders any more. Because you know: Ah, soon it will ring again.

B17

In the session, media support was described as a moderation guide, an information source, and support for the trainers.

It [the in-session slides] should not predominate—to drown out the presentations for example. It should be a guide for the moderation or to visualise something so that you can better understand it, but the dialogue remains central for me.

B21

They used slides, that was...in no sense too much. It took approximately 15-20 minutes. Afterwards we started with tasks or conversing.

B13

Disadvantages mostly referred to certain intervention components with low usability. A prominent critic was concerned by the missing adjustability of mobile app reminders. Some participants suggested that the app’s functionality needed improvement or some extra functions were suggested. In addition, 3 patients remarked that the treatment was not suitable for every patient. Owing to the increased intersession availability, some patients reported a sense of obligation, annoyance, or discouragement as a consequence of the low internet-based compliance. Furthermore, a small number reported suboptimal therapist behavior.

The online platform did not really fit me. I think the principle behind it is okay, but for me it was a kind of obligation.

B1

The app started ringing over and over again and reminded me of all kind of things, which I followed half-heartedly. I like accomplishing things when I’m in the right mood and when I find time.

B12

To use the app several times a day does not help. It only results in additional stress. It would need a kind of surveillance, let’s say, operating without user-input.

B11

Depending on therapist and design, I think [in session] media can also result in more distraction.

B16

### Implications for the Group Setting

#### Facilitating Self-Disclosure and Reinforcement

As contact is usually restricted to the group session, a typical drawback of groups is the limited attention therapists can dedicate to individual patients. Thus, blending brings two important advantages. First, the perceived presence of a therapist (eg, Web-based feedback, support with exercises, or the expression of empathy) can motivate patients to engage in the treatment. Second, some patients described the group setting as a hindrance when it comes to self-disclosure. Here, the extra pathway for the intimate patient-therapist communication constitutes a valued opportunity and provides important information for therapists.

What I liked was the feedback after working on something or after completing an online module. Irena [false name] revised our exercises and gave feedback on it. [...] I think it’s good to have these exercises with a bit of pressure and the feedback system.

B1

It was very important to have home exercises and also daily reminders: Hey, there is something you should work on! For me 70% of the success resulted from keeping up with it; and that it was not only restricted to one day [per week].

B10

There was the story of this football player. I identified with him because I play soccer myself. [...] This was an oppressive situation...Andrea [false name] wrote some very empathic feedback on that. [...]. Based on prior group therapy experience in a psychiatric clinic, I would prefer the blended format because it provides the opportunity to communicate directly with the therapist.

B14

Or the online platform. Here, questions and other things can be answered in detail and in a more direct manner, compared to the group, where shame might be an issue.

B11

Some people might have deep and private issues and they could fear sharing this issues within the group. And there [at the platform], they really can be sure, that it stays with the right person and that they receive some help.

B7

#### Dynamic Interplay of Group and Technology

Besides the improved patient-therapist contact, results suggested a beneficial interplay between the group as a dynamic, self-organizing entity and technology. First, Web-based sessions could provide a preview of subsequent group meetings and thus attune the group to forthcoming themes and activities. Second, a reasonable number of comments indicated smoothing effects on the undesired group dynamics, such as group drifts or domination. Third, in addition to the therapists’ Web-based feedback, group reunions and group support seemed to foster the compliance with Web-based components.

I think it [the structure] was really helpful. It was necessary, [and] nicely implemented. We knew in advance what we were up to in the [next] session. A good solution in every respect.

B6

You spend at least one hour with a topic. [...] So I didn’t say things, I would take back the next moment. You feel more sorted [...] if you listen and look through them [the online exercises]—I suppose a certain structuring happens unconsciously. I would say that’s definitely catalysing.

B19

One participant loved talking and talked a lot. In this situation you have to be concerned about the rest of the group. I would say structure helps in this context. It’s also easier for the trainers.

B21

The previously received [in-session] input gets a good chance [to receive attention]. In this way, a topic was prepared and introduced and then one can comment on it. This facilitates the start of the conversation.

B14

With a short video you can get straight to the point. It’s definitely a big help and a great tool. [...] The explanation at the beginning. The [media supported] meditation exercises are what I liked most.

B12

For me, talking about them [the online exercises] was an extra motivation. Of course you can watch or read everything on your own, but it’s more motivating if you are in a group.

B3

Having the group, and not only the platform and the exercises, was important. That’s what motivated me to do the tasks. [...] Yes, it was the fact, that [...] you get asked in the group.

B5

#### Different Preconditions for Structure and Tailoring

Besides the aforementioned advantages and disadvantages of the blended format per se, blending entails specific implications for the group setting. Even though Web-based and blended interventions allow tailoring to personal needs, individual therapy patients often describe the given predefined treatment structure as restrictive or inflexible. Therefore, we put special emphasis on this issue. Interestingly, the number of patients’ comments on treatment inflexibility or unmet needs was rather low. In this context, trainers’ in-session time management was an issue. However, many patients regarded the structure provided by the format as a guiding thread. In fact, some of the comments suggested different preconceptions and expectations regarding the functional properties of the structure, resulting in (mathematically spoken) inverted signs, when compared with tailoring in individual therapy. As people expect less personal time in groups, blending might even lead to more perceived individuality in group treatments.

It can definitely provide structure, especially in a group setting.

B21

The structure was a real surprise to me. A lot of content, but nicely organised and delivered at the right moment. [...] Some people might ask: What is this? [...] But as the structure progresses, you understand why you did the last exercise.

B22

I would say the structure forced us to come to an end because some patients occupied a lot of time and it did not seem very productive to me. [...] If it is too flexible you go around circles. [...] I don’t think it had to be flexible.

B19

Some things are really good. For example the structure or the “schedule” and to adhere to the “schedule.” [...] It was exactly the same as in the clinic. [...] The transfer into daily life is the hitch and a reminder is what helps.

B18

[...] and it followed a determined procedure. But it was also flexible, so there was not too much content in the sessions and we had time for exchange. [...] It was nicely organised.

B3

Anyway, in the groups it [personal tempo] did not remain in my control, but in the [online] exercises it remained in my hands. And that’s the way it was structured—to have it in my own hands, and to accomplish it the way I want.

B6

I think with depression it [the format] makes sense, because people then have a different motivation to face their problems—but they still receive backing from the group or via the platform, where questions or similar things can be addressed.

B11

I liked the structure. But I had the feeling, we basically always ran out of time. [...] Because there was a big block in the middle, resulting in interposed questions and then you would also like to talk at the end. Sometimes time got a bit thin.

B8

There should have been more time for talking and exchange, as this takes time. [...] I think, “doing things” is the last thing that helps or heals me. I would rather need rest and relaxation.

B20

#### Negative Effects

In the development of new treatments, the occurrence of undesired effects plays an important role. Aside from previously described disadvantages, potential drawbacks concern the negative effects of blended groups on participants’ interaction, trainers’ time, and patient management, as well as dissonance because of the noncompliance with Web-based exercises and low added value of those components. In addition, critical comments were made regarding the design, complexity, and interplay of treatment components, as well as technical issues and data safety. These aspects will be the subject of an upcoming study, focusing on the design and usability issues.

There are these three “pillars [sections]”: the part where we listen and learn, the part where we work at home, and then the interactive part in the group. I think this last part should be expanded.

B7

Maybe it isn’t very personal, because it is only eight sessions.

B22

Some days a bit of extra time for talking and exchange would have done well for me.

B14

I would rather need a “kick in the butt.” The mobile ringing was not enough for me, because I quickly pushed it away [...] and then later when I found time, I had forgotten it already.

B10

This effect [commitment to online exercises] starts vanishing when you don’t finish the task and you realize that it doesn’t have any consequences.

B14

If the group is led poorly and one only relies on online exercises, it can be harmful. The aims for group and online activities should be made very clear because otherwise, I think, people get discouraged. For example, if you work online but it basically has no relevance for the group.

B11

I completed them [the online tasks] and I thought, they were impersonal. There was no connection in terms of “Who reads this?” or “Will it be answered?” [...] Sometimes I felt displaced (upset? unsettled?) when I didn’t complete the exercises.

B2

I frequently experience stress, because I do not get things done. Therefore this [the online exercises] was another potential pitfall for me.

B9

I don’t think it added value. Maybe I’m a little bit old fashioned, but I would have preferred to have it on sheets.

B5

### Summary of Findings

In the synopsis, several important themes emerged from the qualitative analysis, of which some might be addressed by further research. Patients participated for a variety of reasons, but many expressed motives related to the interpersonal exchange and mutual support. bGT was able to elicit surprise and the perception of participating in an unconventional, contemporary, and transparent approach toward treating mental problems. The technology was described as a multiple treatment facilitator. Even though the study does not provide any firm conclusions, it encourages future research on enhanced treatment effects. As expected for effective treatments, bGT resulted in undesired effects such as habituation, dissonance, or overstrain. In addition, signs and reasons for nonresponse were identified. bGT was valued for providing pathways for the intimate patient-therapist communication, which might lead to improvements in the therapeutic relation compared with classic groups. Some patients stated that their motivation to adhere to internet-based and app elements had been fostered by group phenomena. Signs of beneficial effects on undesired group phenomena were found, and the need for personal tailoring seems less relevant in bGT compared with technology-aided individual therapy. No obvious relations existed between assessed patient characteristics (eg, reduction of depressive symptoms; Table 1) and patients’ satisfaction with bGT.

## Discussion

### Principal Findings

This study contributed to the understanding of bGT and added a first qualitative perspective to the existing literature. The qualitative investigation revealed patients’ individual appraisals of the blended Web- and mobile-assisted group treatment and its most salient features. Key findings related to bGT’s positive impact on psychological groups, as well as specific phenomena, categorized by the following themes: dynamic interplay between group and technology, self-disclosure and therapist Web-based guidance, as well as tailoring and structure. Furthermore, results provided information on the possible drawbacks and risks of bGT.

The resulting overall assessment fits the body of the existing literature on blended individual therapy. In line with prior research [21], the flexibility of treatment completion and improved self-management were frequently considered as advantages. In addition, the blended format can be described as a treatment facilitator and motivator, when it comes to between-session support and the transfer into daily life [22]. In accordance with prior studies, bGT was described as a structured and transparent approach to work with depression [25], and we also found evidence for beneficial effects on the therapy process such as improved delivery of evidence-based treatment elements [7]. Last but not least, bGT adds not only therapeutic material to the treatment but also opens new opportunities for (the usually limited) patient-therapist interaction in group therapy.

Many patients described the blended format as a contemporary and versatile approach, which could elicit a positive response and even surprise among its addressees. Thus, bGT can fill an important gap between (pure) Web-based interventions and Web- or mobile-assisted individual therapy. It does so by preserving personal contact at low costs, but—as a modern form of group therapy—it also satisfies the need for interpersonal exchange and relatedness (Theme “A” in Table 2). This feature should be harnessed to develop stigma-free, low-threshold interventions [6,15], which can also be implemented in mental health prevention and stepped care programs [37,38].

### Motivational Agents

Therapist support usually enhances the treatment motivation and outcomes in internet-based therapy [39]. In bGT, however, the therapist is not the only promotor for compliance with these elements [40]. Instead, anticipated group reunions and in-session discussions foster the motivational momentum. This represents a novel finding in blended therapy, which might be addressed by further investigation. In addition, these findings add a new aspect to the classical literature on therapeutic group factors. The prior group therapy literature proposes merely positive agents such as interpersonal learning, imparting of information [41], and guidance [42]; these factors have consistently been related to the treatment outcome in more recent group therapy studies [43]. Regarding the compliance with internet-based homework, some patients reported motivational agents, which better fit phenomena rooted in social psychology—for example, the “Challenge and threat” or the “Learned drive” hypotheses of social facilitation theory [44,45]. Thus, on the micro level, certain aversive agents seem addable to the interplay of predominantly positive concepts, known from the classical research on therapeutic group factors [41,42], or from the literature on SDT in blended care [23,24].

### Treatment Transparency and Reduction of Drift-offs

In this study, patients appreciated the transparency and increased salience of the treatment, as well as its availability between single sessions. In this context, Mansson et al [7] described the beneficial effects of the technology support on undesired drift-offs from postulated optimal CBT rationales [26]. We intend to further differentiate this finding by suggesting a “double function of alignment.” On one hand, the between-session computer and app support can provide a dramaturgical frame, allowing patients to autonomously prepare, rework, or deepen the content of a given session, resulting in an optimization of between-session processes. On the other hand, in-session media help therapists establish a guiding roadmap through a given session. The technology-based in-session support, therefore, furthers allegiance beyond those blended rationales, which provide only the between-session support [7]. Regarding psychological groups (or difficult patient populations), this can also result in the reduction of undesired interactional processes, especially the therapist drift, group drift, or domination.

### Self-Disclosure and Internet-Based Reinforcement

Self-disclosure is an important, but also sensitive, subject in psychological groups [46]. The choice of intimate patient-therapist communication (through the platform and in the absence of other group members) can support this sensitive process and constitutes a novel pathway for self-disclosure. It simultaneously counteracts a main risk factor for adverse outcomes in group therapy—being conflicted about self-disclosure and intimacy [47]. Furthermore, many patients valued the received personal feedback on accomplished CBT tasks. Consequently, blending has the potential to blur distinctions between individual and group therapy by facilitating seamless shifts between both modalities.

### Diverging Preconditions

Despite the coherent findings with other forms of blended therapy, certain preconditions and phenomena differ in the bGT paradigm. First, the adaptability to personal needs is a prominent challenge for internet-based and blended therapy [5,13]. As a matter of fact, psychological groups have to follow a consensual course, and participants agree to this situation when participating. On a basic level, one of the challenges of internet-based and blended treatments (tailoring) seems to play a minor role in bGT. Furthermore, the appraisal of technology-induced structure seems to shift from a reported restriction in blended individual therapy to a consensual guiding thread in psychological groups.

### Negative Effects of Blending

Besides the beneficial effects, computer and app support might also be accompanied by undesired effects. In this study, the possible negative effects can be categorized into effects on the patient, group, and therapist levels. Regarding negative effects on individual patients, the spectrum ranges from hassles because of the improvable usability or technical issues up to the discouragement elicited by the noncompliance with internet-based tasks. As homework adherence often relates to the treatment success [48,49], therapists should examine whether the noncompliance is caused by doubts in the treatment rationale or whether it rather constitutes typical disease symptoms. For the latter, the therapist feedback system offers new opportunities to individually monitor and reinforce the adherence. In this context, we also found signs of suboptimal therapist behavior. Some therapists occasionally failed to provide internet-based feedback, leading to patients losing some treatment motivation. In addition, the successful in-session media management, in terms of time management and balancing of modalities (eg, psychoeducation vs discussion or exercising), is an art in itself. As blended interventions require therapists to manage various dramaturgical threads in parallel, extensive training, practical experience, and reimbursement of trainers’ internet-based time help to optimize the course of treatment. Nonetheless, further research on therapists’ evaluation of bGT is warranted. As a final aspect, blending might excerpt a undesired influence on the group as an entity. In line with prior findings, very short treatments might not always be preferred by patients [15], as they restrict the time patients need to familiarize and establish trust [50]. If a given treatment is planned to be short in duration, the intervention can be advertised as modern psychological training rather than classic group psychotherapy. This relational frame can help increase curiosity about the treatment, might reduce possible stigmatization or false expectations, and also offer interesting prospects for low resource prevention and stepped care. However, independent of the overall duration, sufficient in-session time for group exercises and discussions should be foreseen.

### Strengths and Limitations

This study exhibits a variety of noteworthy strengths and limitations. Among its most important strengths, this study builds upon a high number of in-depth interviews. In addition, the proportion of patients participating in both the intervention and the interviews was high. Furthermore, we were able to depict more critical patient views by including withdrawals, deteriorations, and relatively unsatisfied patients (by means of standardized measures, such as SUS and CSQ). Regarding the data analysis, 3 psychologists were included in the standardized procedure, resulting in a high level of mutual control. Finally, each intervention has setting- and design-specific properties, resulting in specific patient experiences. Including patients from two different interventions, therefore, increases the generalizability of presented results, which especially applies as both subsamples were represented in comparable parts.

However, this study also has noteworthy limitations. First, the investigated sample was self-selected, and even though demographic characteristics of the original studies (eg, comorbidity, education, and gender) seem to reflect patients’ properties in routine care, the interventions took place in a university outpatient setting with affiliated and well-trained therapists or student therapists. Therefore, the suboptimal therapist behavior might occur more frequently in routine care. In addition, patients in this study might have been more motivated and compliant than those in routine practice. Second, even though we were able to include a variety of experiences, negative selection might have occurred in the absence of extremely critical or very disappointed patients. While the spectrum of experienced system usability (SUS) was depicted exhaustively, a minor selection bias might have occurred with clients exhibiting strong overall service dissatisfaction (eg, double “✗” on CSQ). Third, the time of assessment varied widely, ranging as far as up to 9 months after participation. Thus, as much as a quarter of data might reflect rather distant treatment memories, which tend to be less differentiated and also have an increased risk of memory distortions, compared with those of more recent interviewees. Fourth, interviews were conducted in equal parts by the first and second authors (RS and SS), introducing an increased risk of bias. To counteract this tendency, patients were encouraged to freely share their views (eg, pointing out the value of critical views), and standardized protocols were set up beforehand (eg, evaluation by a third analyst, not affiliated to bGT research). Furthermore, both interviewers closely adhered to the interview guide, resulting in a high convergence between the applied interview guide (Textbox 1 and Multimedia Appendix 1) and gained themes (Table 2). Fifth, this study only presents patients’ experiences but not those of participating therapists. In this respect, the corresponding information on blended individual therapy is provided in the recent literature [51]. Regarding bGT, a forthcoming study on the therapist-centered feasibility of bGT is currently being prepared by our research group. Sixth, obtained data were not systematically analyzed with respect to subsamples (eg, intervention A vs B or patient subgroups). Even though the information from Table 1 does not suggest any obvious relation between the given subgroups (eg, the relation between depressiveness and system usability or the relation between a given intervention and service satisfaction), this study could have benefited from purposive sampling. In this context, a prior investigation into one of the subsamples (Intervention A) failed to detect such relations [6]. However, we are currently accumulating data over several trials to investigate potential predictors with sufficient statistical power.

### Future Directions

This is the first qualitative study that supports prior findings on bGT for depression [6,15,38,40] and opens new questions to be addressed in future. First, in order to estimate the impact of bGT on observable treatment effects, this novel approach should be tested for its clinical effectiveness in future studies [14,16]. Second, as bGT was perceived as a contemporary, low-threshold approach with a character similar to training [15], its merits for the prevention of mental health disorders should be tested. Third, many possible arrangements emerge from the combination of internet and group elements, but only a few have been investigated so far. For example, bGT can blur distinctions between individual and group therapy and therefore might be a valuable option for settings in which a hybrid form of both modalities is desired. Furthermore, the combination of bGT with tele-groups should be tested as a location-independent version of bGT. Fourth, previously mentioned side effects of bGT need to be addressed through future research. In light of this, the implications of providing a classic face-to-face therapy path for less interested patients could be investigated. Fifth, gained results suggest the beneficial effects of lateral communication in bGT on therapeutic alliance. This assumption could be tested by applying corresponding measures (eg, Working Alliance Inventory) in comparative trials [52]. Sixth, the reported effects on group phenomena need to be investigated more objectively. Here, the group interaction could be videotaped and analyzed by means of automatized video software (eg, Mobile Event App). Finally, the presented bGT depression studies have been conducted in a university outpatient setting. As results have been found to be promising, bGT should be tested in standard care.

### Conclusions

Taken together, the results from in-depth interviews underpin previous findings on the feasibility of bGT for depression. The innovative format contributes to a participatory and proactive treatment of depression and may also be applied in depression prevention. In addition, Web- and mobile-assisted groups yield beneficial modalities and phenomena, increasing the treatment quality and addressing some common disadvantages of classic group therapy. However, potential drawbacks need to be considered carefully, and interventions should always be tailored to the needs of all three—involved patients, therapists, and institutions. Owing to the novelty of this approach, further research on the perspectives of involved therapists and stakeholders is warranted.

## Abbreviations

ACT: Acceptance and Commitment Therapy
bGT: blended group therapy
CBT: cognitive behavioral therapy
CSQ: Client Satisfaction Questionnaire
MDD: Major depressive disorder
SDT: Self-Determination Theory
SUS: System Usability Scale

## Appendix

Multimedia Appendix 1 Interview guide.

## References

[1] Vigo D, Thornicroft G, Atun R (2016). Estimating the true global burden of mental illness. The Lancet Psychiatry.

[2] Wykes T, Haro JM, Belli SR, Obradors-Tarragó Carla, Arango C, Ayuso-Mateos JL, Bitter I, Brunn M, Chevreul K, Demotes-Mainard J, Elfeddali I, Evans-Lacko S, Fiorillo A, Forsman AK, Hazo J, Kuepper R, Knappe S, Leboyer M, Lewis SW, Linszen D, Luciano M, Maj M, McDaid D, Miret M, Papp S, Park A, Schumann G, Thornicroft G, van DFC, van OJ, Wahlbeck K, Walker-Tilley T, Wittchen H, ROAMER consortium (2015). Mental health research priorities for Europe. Lancet Psychiatry.

[3] Johansson R, Andersson G (2012). Internet-based psychological treatments for depression. Expert Rev Neurother.

[4] Richards D, Richardson T (2012). Computer-based psychological treatments for depression: a systematic review and meta-analysis. Clin Psychol Rev.

[5] Andersson G, Titov N (2014). Advantages and limitations of Internet-based interventions for common mental disorders. World Psychiatry.

[6] Schuster R, Leitner I, Carlbring P, Laireiter A (2017). Exploring blended group interventions for depression: Randomised controlled feasibility study of a blended computer- and multimedia-supported psychoeducational group intervention for adults with depressive symptoms. Internet Interventions.

[7] Månsson Kristoffer N T, Skagius RE, Gervind E, Dahlin M, Andersson G (2013). Development and initial evaluation of an Internet-based support system for face-to-face cognitive behavior therapy: a proof of concept study. J Med Internet Res.

[8] Craske MG, Rose RD, Lang A, Welch SS, Campbell-Sills L, Sullivan G, Sherbourne C, Bystritsky A, Stein MB, Roy-Byrne PP (2009). Computer-assisted delivery of cognitive behavioral therapy for anxiety disorders in primary-care settings. Depress Anxiety.

[9] Wright JH, Wright AS, Albano AM, Basco MR, Goldsmith LJ, Raffield T, Otto MW (2005). Computer-assisted cognitive therapy for depression: maintaining efficacy while reducing therapist time. Am J Psychiatry.

[10] Romijn G, Riper H, Kok R, Donker T, Goorden M, van RLH, Kooistra L, van BA, Koning J (2015). Cost-effectiveness of blended vs. face-to-face cognitive behavioural therapy for severe anxiety disorders: study protocol of a randomized controlled trial. BMC Psychiatry.

[11] Kleiboer A, Smit J, Bosmans J, Ruwaard J, Andersson G, Topooco N, Berger T, Krieger T, Botella C, Baños Rosa, Chevreul K, Araya R, Cerga-Pashoja A, Cieślak R, Rogala A, Vis C, Draisma S, van SA, Kemmeren L, Ebert D, Berking M, Funk B, Cuijpers P, Riper H (2016). European COMPARative Effectiveness research on blended Depression treatment versus treatment-as-usual (E-COMPARED): study protocol for a randomized controlled, non-inferiority trial in eight European countries. Trials.

[12] Kooistra LC, Wiersma JE, Ruwaard J, van OP, Smit F, Lokkerbol J, Cuijpers P, Riper H (2014). Blended vs. face-to-face cognitive behavioural treatment for major depression in specialized mental health care: study protocol of a randomized controlled cost-effectiveness trial. BMC Psychiatry.

[13] Erbe D, Eichert H, Riper H, Ebert DD (2017). Blending Face-to-Face and Internet-Based Interventions for the Treatment of Mental Disorders in Adults: Systematic Review. J Med Internet Res.

[14] Zwerenz R, Becker J, Knickenberg RJ, Siepmann M, Hagen K, Beutel ME (2017). Online Self-Help as an Add-On to Inpatient Psychotherapy: Efficacy of a New Blended Treatment Approach. Psychother Psychosom.

[15] Schuster R, Fichtenbauer I, Sparr VM, Berger T, Laireiter A (2018). Feasibility of a blended group treatment (bGT) for major depression: uncontrolled interventional study in a university setting. BMJ Open.

[16] Berger T, Krieger T, Sude K, Meyer B, Maercker A (2018). Evaluating an e-mental health program (“deprexis”) as adjunctive treatment tool in psychotherapy for depression: Results of a pragmatic randomized controlled trial. J Affect Disord.

[17] (2009). National Institute for Health and Care Excellence.

[18] Gruber K, Moran PJ, Roth WT, Taylor CB (2001). Computer-assisted cognitive behavioral group therapy for social phobia. Behavior Therapy.

[19] Newman MG, Przeworski A, Consoli AJ, Taylor CB (2014). A randomized controlled trial of ecological momentary intervention plus brief group therapy for generalized anxiety disorder. Psychotherapy (Chic).

[20] Przeworski A, Newman MG (2004). Palmtop computer-assisted group therapy for social phobia. J Clin Psychol.

[21] van DVR, Witting M, Riper H, Kooistra L, Bohlmeijer ET, van GLJ (2014). Blending online therapy into regular face-to-face therapy for depression: content, ratio and preconditions according to patients and therapists using a Delphi study. BMC Psychiatry.

[22] Ly KH, Janni E, Wrede R, Sedem M, Donker T, Carlbring P, Andersson G (2015). Experiences of a guided smartphone-based behavioral activation therapy for depression: A qualitative study. Internet Interventions.

[23] Wilhelmsen M, Lillevoll K, Risør M, Høifødt R, Johansen M, Waterloo K, Eisemann M, Kolstrup N (2013). Motivation to persist with internet-based cognitive behavioural treatment using blended care: a qualitative study. BMC Psychiatry.

[24] Ryan RM, Deci EL (2000). Self-determination theory and the facilitation of intrinsic motivation, social development, and well-being. American Psychologist.

[25] Lillevoll KR, Wilhelmsen M, Kolstrup N, Høifødt RS, Waterloo K, Eisemann M, Risør MB (2013). Patients' experiences of helpfulness in guided internet-based treatment for depression: qualitative study of integrated therapeutic dimensions. J Med Internet Res.

[26] Waller G (2009). Evidence-based treatment and therapist drift. Behav Res Ther.

[27] Hayes SC, Strosahl KD, Wilson KG (2012). Acceptance and Commitment Therapy: The Process and Practice of Mindful Change.(2nd edition). Acceptance and Commitment Therapy: The Process and Practice of Mindful Change.

[28] Jacobson N, Martell C, Dimidjian S (2006). Behavioral activation treatment for depression: Returning to contextual roots. Clinical Psychologycience and Practice.

[29] Minddistrict W minddistrict.

[30] Margraf J (2013). Mini-DIPS: Diagnostisches Kurz-Interview bei Psychischen Störungen. Mini-DIPS: Diagnostisches Kurz-Interview bei Psychischen Störungen.

[31] Larsen DL, Attkisson C, Hargreaves WA, Nguyen TD (1979). Assessment of client/patient satisfaction: Development of a general scale. Evaluation and Program Planning.

[32] Brooke J, Jordan PW, Thomas B, Weerdmeester BA, McClelland AL (1996). Usability Evaluation in Industry. SUS: a “quickdirty” usability scale.

[33] Hautzinger M, Bailer M (1993). Allgemeine Depressions-Skala. Allgemeine Depressions-Skala.

[34] Braun V, Clarke V (2006). Using thematic analysis in psychology. Qualitative Research in Psychology.

[35] Fereday J, Muir-Cochrane E (2016). Demonstrating Rigor Using Thematic Analysis: A Hybrid Approach of Inductive and Deductive Coding and Theme Development. International Journal of Qualitative Methods.

[36] Mayring P, Fenzl T (2018). QCAmap.

[37] Sander L, Rausch L, Baumeister H (2016). Effectiveness of Internet-Based Interventions for the Prevention of Mental Disorders: A Systematic Review and Meta-Analysis. JMIR Ment Health.

[38] Delgadillo J, Kellett S, Ali S, McMillan D, Barkham M, Saxon D, Donohoe G, Stonebank H, Mullaney S, Eschoe P, Thwaites R, Lucock M (2016). A multi-service practice research network study of large group psychoeducational cognitive behavioural therapy. Behav Res Ther.

[39] Berger T (2017). The therapeutic alliance in internet interventions: A narrative review and suggestions for future research. Psychother Res.

[40] Aguilera A, Bruehlman-Senecal E, Demasi O, Avila P (2017). Automated Text Messaging as an Adjunct to Cognitive Behavioral Therapy for Depression: A Clinical Trial. J Med Internet Res.

[41] Yalom I, Leszcz M (2005). The Theory and Practice of Group Psychotherapy. 5th ed. The Theory and Practice of Group Psychotherapy. 5th ed.

[42] Bloch S, Crouch E (1985). Therapeutic Factors in Group Psychotherapy. Therapeutic Factors in Group Psychotherapy.

[43] Kivlighan DM, Conyne B, Miles, Paquin (2010). Therapeutic factors in group counseling: Asking new questions. The Oxford handbook of group counselling.

[44] Blascovich J, Mendes WB, Hunter SB, Salomon K (1999). Social “facilitation” as challenge and threat. Journal of Personality and Social Psychology.

[45] Strauss B (2002). Social facilitation in motor tasks: a review of research and theory. Psychology of Sport and Exercise.

[46] Burlingame GM, Fuhriman A, Johnson JE (2001). Cohesion in group psychotherapy. Psychotherapy: Theory, Research, Practice, Training.

[47] Roback HB (2000). Adverse outcomes in group psychotherapy: risk factors, prevention, and research directions. J Psychother Pract Res.

[48] Conklin LR, Strunk DR (2015). A session-to-session examination of homework engagement in cognitive therapy for depression: Do patients experience immediate benefits?. Behav Res Ther.

[49] Kazantzis N, Whittington C, Dattilio F (2010). Meta-analysis of homework effects in cognitive and behavioral therapy: A replication and extension. Clinical Psychology: Science and Practice.

[50] Forsyth D (2013). Group Dynamics. 6th ed. Group Dynamics. 6th ed.

[51] Titzler I, Saruhanjan K, Berking M, Riper H, Ebert DD (2018). Barriers and facilitators for the implementation of blended psychotherapy for depression: A qualitative pilot study of therapists' perspective. Internet Interventions.

[52] Andersson G, Paxling B, Wiwe M, Vernmark K, Felix CB, Lundborg L, Furmark T, Cuijpers P, Carlbring P (2012). Therapeutic alliance in guided internet-delivered cognitive behavioural treatment of depression, generalized anxiety disorder and social anxiety disorder. Behav Res Ther.

