# Supplementary material for: Patients’ Experiences of Web- and Mobile-Assisted Group Therapy for Depression and Implications of the Group Setting: Qualitative Follow-Up Study
Source: JMIR Ment Health. 2018 Jul 11;5(3):e49. doi: 10.2196/mental.9613 (PMC6060305; doi:10.2196/mental.9613)
Supplement: Multimedia Appendix 1 [file mental_v5i3e49_app1.pdf]

Appendix A

Interview guide

1. What were your expectations regarding the treatment?
(Supplementary questions: Why did you participate? What was important to you?)
2. How would you describe the treatment?
(Supplementary questions: What do you think of the treatment? Do you have any positive or negative memories?)
3. How would you describe the treatment’s format (comprising group and media components)? (Supplementary question: How was your experience with the online (and app) components?)
4. How did you use the online platform and the videos? … the app?
How did you experience their usability?
5. How did you experience the group? In your view, what are particular advantages and disadvantages of the group setting?
- What did you experience positively?
- What did you experience negatively?
- What would you do differently if you could?
6. In your view, what are the particular advantages and disadvantages of the computer-supported / app-supported format?
- What did you experience positively?
- What did you experience negatively?
- Did you experience any problems?
- In your opinion, what tasks are those formats suitable for?
7. Regarding modern media (app + platform + in-session), what would you change if you could?
8. How would you evaluate the blended format?
(Supplementary question: When does/doesn’t it make sense to apply this format?
9. How did technology influence the group / group interaction?
10. *There was a lot of predefined content in the treatment (e.g. PowerPoint presentations during sessions or online videos).* How would you describe the treatment in terms of structure and flexibility?
11. How did the therapists handle technology?
(Supplementary questions: To what extent did technology support or hinder involved therapists? How would you describe the blended format from the viewpoint of a therapist?)
12. How useful is the application of modern media in psychological groups?
(Supplementary questions: From your point of view, how meaningful is the application of modern media in such treatments? Why?)
13. How would you describe the treatment’s intensity?
14. Was there anything particularly difficult to motivate yourself for? Was it difficult to motivate yourself for the group or the online tasks?
(Supplementary questions: What motivated / demotivated you? Were you able to find time for the group or online tasks in your daily life?)
15. What influence did the intervention have on your thoughts, feelings, plans and behaviour? Why did the intervention have this positive or negative impact?

16. Is there anything (important) you would like to add to this conversation? (Supplementary questions: Was there anything you missed after the treatment had ended? … anything more you would have needed?)
